# Local patterns of diversity in California northern coastal scrub

**DOI:** 10.1002/ece3.4104

**Published:** 2018-06-27

**Authors:** Eric Wrubel, V. Thomas Parker

**Affiliations:** ^1^ Department of Biology San Francisco State University San Francisco California

**Keywords:** coastal, diversity, environmental gradient, hotspot, shrublands, vegetation

## Abstract

Within global biodiversity hotspots such as the California Floristic Province, local patterns of diversity must be better understood to prioritize conservation for the greatest number of species. This study investigates patterns of vascular plant diversity in relation to coast–inland environmental gradients in the shrublands of Central California known as northern coastal scrub. We sampled coastal shrublands of the San Francisco Bay Area at coastal and inland locations, modeled fine‐scale climatic variables, and developed an index for local exposure to maritime salts. We compared diversity, composition, and structure of the coastal and inland plots using indirect gradient analysis and estimated species accumulation using rarefaction curves. Coastal plots were significantly higher in alpha, beta, and gamma diversity than inland plots. Plant diversity (effective species number) in coastal plots was 2.1 times greater than inland plots, and beta diversity was 1.9 times greater. Estimated richness by rarefaction was 2.05 times greater in coastal sites than inland sites. Salt deposition and water availability were the abiotic process most strongly correlated with increased maritime plant diversity and compositional differences. Stands of northern coastal scrub on the immediate coast with higher maritime influence exhibit markedly higher plant diversity than most interior stands, paralleling previous work in other vegetation types in this region. These studies suggest that the California coastline deserves special consideration for botanical conservation. Fine‐scale climatic models of cloud frequency, water availability, and the salt deposition index presented here can be used to define priority areas for plant conservation in California and other coastal regions worldwide.

## 1 INTRODUCTION

The spatial distribution of biological diversity and its determinants have long been a primary concern for ecologists and biogeographers, with significant modern applications in ecological theory and conservation biology (Ferrier & Drielsma, 2010; Gaston, 2000). Delineation and conservation of global biodiversity hotspots have gained particular significance as anthropogenic impacts have increased (Brooks et al., 2006; Mittermeier, Turner, Larsen, Brooks, & Gascon, 2011; Myers, Mittermeier, Mittermeier, da Fonseca, & Kent, 2000). The 35 designated global biodiversity hotspots cover only 17.3% of earth's land surface, yet contain over 50% of the world's plant species as endemics (Mittermeier et al., 2011; Williams et al., 2011). Within these priority areas, however, smaller‐scale patterns of richness and endemism often are less well understood (Kremen et al., 2008). This represents a significant conservation knowledge gap because many land use decisions are made at the local scale by regional governments, agencies, and private landholders. The relationship between local patterns of diversity and local environmental gradients is only well studied in a few systems, like tidal marshes and vernal pools, but is not yet understood well enough to build predictive models across landscapes. Effective policy governing protection and management of local biodiversity hotspots require understanding smaller‐scale patterns of diversity along environmental gradients.

The California Floristic Province (CFP) is one of the five Mediterranean‐type climate zones (MTCs) worldwide, each is among the 35 global biodiversity hotspots, areas of exceptionally high plant diversity and endemism (Burge et al., 2016; Myers et al., 2000). Regional patterns of plant diversity in MTCs may be explained both by phylogenetic analysis, in relation to historic environmental change, and by ecological analysis, in relation to environmental factors influencing contemporary species coexistence (Linder, 1991). Explosive Quaternary species radiations occurred in all MTCs, in response to drought, fire, and other stressors associated with aridification, while low extinction rates or radiations of older lineages were associated with refugia from these climatic extremes or refugia from cooling and glaciation during ice ages (Ackerly, 2009; Cowling & Lombard, 2002; Keeley & Swift, 1995; Kraft, Baldwin, & Ackerly, 2010; Raven & Axelrod, 1978; Rundel et al., 2016; Stebbins & Major, 1965). Contemporary patterns of plant diversity in MTCs have been shown to be positively correlated with environmental heterogeneity in soils, topography, precipitation, and temperature (Casazza, Zappa, Mariotti, Médail, & Minuto, 2008; Harrison, Viers, & Quinn, 2008; Linder, 1991; Richerson & Lum, 1980). Disturbance factors including fire and herbivory may be positively or negatively correlated with contemporary plant diversity in MTCs (Harrison, Inouye, & Safford, 2003). Species coexistence at the regional scale is maintained by environmental factors and processes, which can be identified to generate predictive spatial models of diversity hotspots or coldspots and applied to conservation actions for multiple taxa.

Within the CFP, plant diversity is heterogeneously distributed, concentrated in the Sierra Nevada foothills and the Coast Ranges, and especially in the central part of the outer Coast Ranges (Central West region), a mountainous coastal area from the San Francisco Bay Area south toward Point Conception (Burge et al., 2016; Kraft et al., 2010). In the Central West region, a general phenomenon is that plant diversity in many vegetation types is concentrated along the coast. A query of herbarium records from subregions within the Central West bioregion (CCH, 2015) shows that richness of minimum‐rank plant taxa in the narrow coastal subregion (Central Coast) is 4.1 times greater per ha than the much larger adjacent interior subregions of the San Francisco Bay Area and South Coast Ranges (Baldwin et al., 2012). Several studies have shown plant diversity in CFP communities to be positively correlated with proximity to the coast. Species richness of edaphic endemics on serpentine soils was higher at sites closer to the coast and declined at interior sites in Northern and Central California (Harrison et al., 2008), as did richness of Central California coastal prairies compared to interior grasslands (Stromberg, Kephart, & Yadon, 2001). Similarly, beta diversity in chaparral associations in Central California was higher among sites associated with coastal fog than at interior sites associated with greater continentality (Vasey, Parker, Holl, Loik, & Hiatt, 2014).

As a test of this coastal diversity pattern, here we investigate patterns of plant diversity in relation to environmental gradients within the coastal shrublands of Central California, collectively referred to as northern coastal scrub (Ford & Hayes, 2007; Munz & Keck, 1959; Sawyer, Keeler‐Wolf, & Evens, 2009). Northern coastal scrub (NCS) is a dominant vegetation type of coastal hills and plains in Central California, ranging from Santa Barbara to the Oregon border and inland to the Sierra foothills, wherever maritime influence moderates climatic extremes. NCS exhibits a wide range in composition and structure along gradients of aridity, maritime influence, and topographic position. Coyote brush (*Baccharis pilularis* DC.) is characteristically dominant or codominant. This evergreen, salt‐tolerant shrub exhibits variable habit, with prostrate to upright forms from 0.25 to 4 m height. Some NCS associations have a well‐developed herbaceous layer and are exceptionally diverse in vascular plant species and life forms, while others are dominated by relatively few, large shrub species, with a sparser, less diverse herbaceous layer. One hundred and six rare or endangered plant taxa are associated with NCS vegetation, of which 79 are California endemics (Calflora, 2015; CNPS, 2015). Despite its floristic and physiognomic diversity and its recognition as a major shrubland formation of the CFP (Westman, 1981), relatively little formal study of NCS has occurred.

While California's coastal zone harbors a significant percentage of the plant diversity within the CFP, it is also heavily impacted and threatened by altered ecological processes due to development (Tang, 2008), invasive species (Dukes & Mooney, 2004), and climate change (Ackerly et al., 2010). Our objectives are to assess patterns of diversity within NCS as a first step to conservation efforts. We hypothesized that patterns of diversity in NCS would be similar to other vegetation types in the region, with most diversity concentrated closest to the coast. In particular, we expected NCS would exhibit a local hotspot pattern of highest diversity in a narrow zone near the edge of coastal bluffs and headlands. We consequently also consider the correlation of prominent environmental factors with diversity patterns as a way of understanding processes sustaining diversity in this vegetation. These include soil moisture and aerosol salinity as well as several additional edaphic and climatic influences. Effective conservation in this, and other coastal regions worldwide, will require higher resolution information on the patterns of biodiversity and environmental correlation, to identify the areas and factors necessary to protect the greatest numbers of taxa from extinction.

## 2 METHODS

### 2.1 Study area and sample design

**Figure 1:**
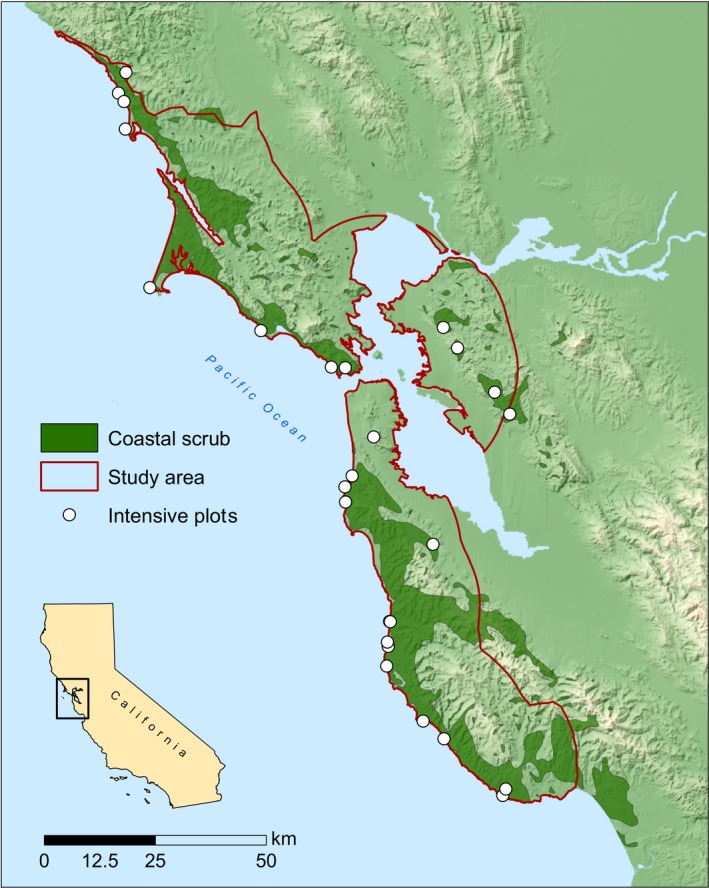
The study area, outlined in red, within the San Francisco Bay Area bioregion, extending from the coast to 20 km inland. The extent of northern coastal scrub vegetation in the region is shown in dark green. Intensive sample plot locations are displayed as white dots

The study area was within the San Francisco Bay Area bioregion (Baldwin et al., 2012) and extended from the coast to 20 km inland, the range of northern coastal scrub in the bioregion (Figure 1). We sampled a total of 114 plots in two phases, an initial intensive sampling phase, and a second rapid assessment phase. In the intensive sampling phase, a small number of sample sites were randomly selected and sampled extensively for vegetation and environmental attributes in order to analyze vegetation structure and composition in relation to environmental variables. In the rapid assessment phase, a larger number of sites were sampled for fewer attributes along gradient‐directed transects (Gillison & Brewer, 1985). The purpose of the second sampling phase was to capture a greater range of species associations in NCS vegetation in order to classify the associations into functional groups.

### 2.2 Intensive sampling

Within the study area, we used NCS vegetation polygons mapped in the coyote brush and California sagebrush (*Artemisia californica*) vegetation alliances in Calveg (USDA, 2003) to delineate the sample area (Figure 1). We then stratified the sample area into coastal bluff and inland zones. The coastal bluff zone was comprised of sites at the edge of coastal cliffs above the shoreline, between 0 and 0.5 km from the coast. The inland zone was between 0.5 and 20 km inland from the coast. We randomly located 18 sample points in the coastal bluff zone and nine points in the inland zone. The coastal bluff zone had twice as many points as the inland zone because we initially stratified a middle zone between 0.25 and 0.5 km from the coast, but eventually merged this into a single coastal bluff zone between 0 and 0.5 km from the coast, due to the highly heterogeneous topography of the coastline. Plots were selected that contained >30% woody plant cover and had no evidence of recent anthropogenic disturbance, livestock grazing, or fire.

At each sample point, we placed a 400 m^2^ plot measuring 20 × 20 m. Within the plot, we estimated percent cover (Daubenmire, 1968) of all minimum‐rank vascular plant taxa. Minimum‐rank taxa are hereafter referred to as species. Nomenclature followed the Jepson Manual (Baldwin et al., 2012). We collected and analyzed soil samples in 17 coastal bluff plots and seven inland plots. We collected four soil samples per plot to 6 cm below the surface, at the center of each quadrant, and analyzed them for % organic matter, pH, and mineral nutrients (N, P, K, Ca, S, Mg, and Na; Table S2). These analyses were performed at Western Agricultural Laboratories, Modesto, California. Soil texture was characterized using the method of Brewer and McCann (1982).

### 2.3 Rapid assessment sampling

In the second sampling phase, 87 plots were located along transects, utilizing gradient‐directed sampling (Gillison & Brewer, 1985; Parker, Schile, Vasey, & Callaway, 2011). The goal was to rapidly sample numerous NCS stands in order to classify different species associations into functional groups, to better interpret the results of the intensive sampling. We restratified the study area into three zones (north, central, and south) and located two transects in each zone, one transect close to the coast and one transect inland. The transects followed trails running through NCS vegetation and averaged 2 km in length. Transects were located along routes with heterogeneous topographic exposure, and varying aspect and elevation, to sample the high vegetation turnover we observed along local environmental gradients. We sampled progressively along each transect, placing plots in each new stand encountered that was a coastal scrub vegetation association. The plots were placed in relatively heterogeneous vegetation representative of the whole stand, following standard sampling procedures to classify vegetation alliances and associations (CNPS, 2007). The plots measured 400 m^2^ and were placed at least 10 m from the edge of the trail. There were roughly 14 plots per transect. In each plot, we estimated percent cover for all tree and shrub species and at least three dominant species in the herb layer and recorded slope, aspect, and coordinates. The goal of this sampling effort was to gather data on the composition of various NCS species associations to create a vegetation classification that would aid interpretation of the intensive sampling results. A list of all vascular plant taxa observed in both sampling phases is provided in Table S1.

### 2.4 Overlay analysis

**Figure 2:**
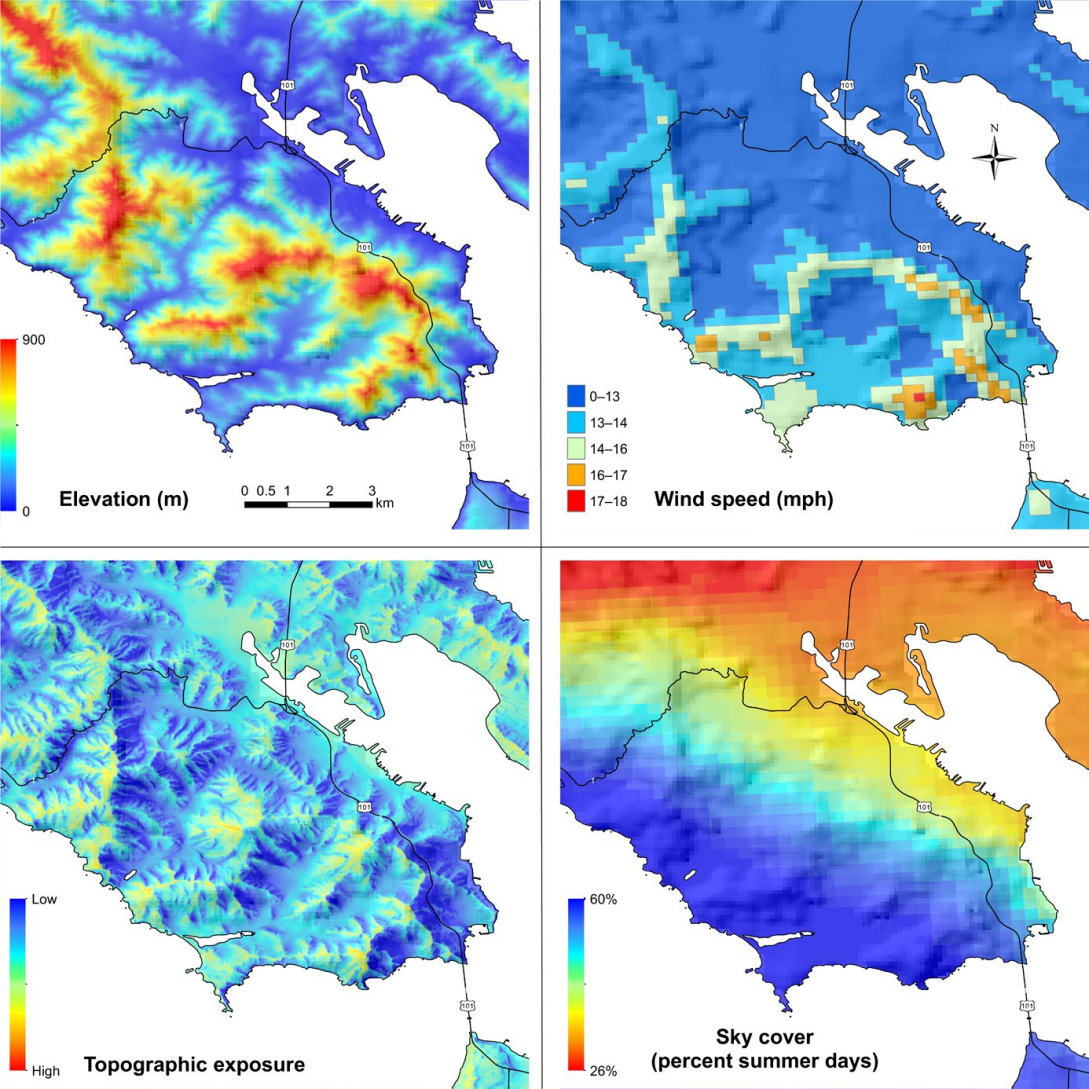
Topographic and climatic overlays for Sausalito, California: elevation (upper left); wind speed (upper right); index of topographic exposure to prevailing winds or topex (lower left); frequency of summertime cloud cover or sky cover (lower right)

Environmental variables that were not measured directly in the field were extrapolated to the intensive plot coordinates using GIS (Figure 2, Figures [Link], [Link], [Link], [Link], [Link]). We measured the distance to coast of each plot. We estimated mean annual precipitation, maximum temperature of the warmest month, and minimum temperature of the coldest month at each site, at a resolution of 1 km^2^ (WorldClim gridded climate dataset, Hijmans, Cameron, Parra, Jones, & Jarvis, 2005); mean annual wind speed at a resolution of 200 m^2^ (NREL, 2003); and heat load at the plot coordinates (McCune & Keon, 2002). Cloud frequency was acquired from a composite of MODIS satellite images between July and October from 2000 to 2006, giving the mean frequency of days with cloud cover at 10:00 a.m. (Fischer, Still, & Williams, 2009). We modeled topographic wind exposure (topex) for each intensive sample site, using a 30‐m resolution digital elevation model (DEM; USGS, 2012). Topex indexes the topographic modification of local wind speeds at a given location (Wilson, 1984). We used a topex‐to‐distance of 2 km (Quine & White, 1994).

### 2.5 Salt deposition modeling

We developed an index for local exposure to maritime salts that integrates previously established equations for coastal salt deposition, vertical salt distribution in the troposphere, and topographic exposure. We integrated a coastal salt transportation and deposition formula (equation (1); Meira, Andrade, Padaratz, Alonso, & Borba, 2006; Meira, Andrade, Alonso, Padaratz, & Borba, 2007) with an expression for the vertical concentration gradient of salts in maritime air masses (Equation (2); Blanchard & Woodcock, 1980). The sum of these two formulas gives an estimate of salt deposition for terrestrial locations at a given distance from the coast, a given elevation above sea level, and a given wind speed. The degree to which wind exposure is modified by topography at a given site is expressed by multiplying the deposition functions by the topex score.

(1) $$D=D_{0}\exp^{\left(\exp(-x/w)-1\right)}$$

(2) $$S=5{\left(6.3∙10^{-6}H\right)}^{\left(0.21-0.39\log w\right)}$$

(3) y=D(1−T)+S(1−T)

Equation (1) gives *D* as the dry deposition of salts (mg m^−2^ s^−1^) at a distance *x* (km) from the coast; *D*_0_ as the dry deposition of salts at the shoreline; and *w* as the wind speed (m/s). Equation (2) gives *S* as the average sea‐salt concentration in an air mass (μg/m^3^); and *H* as the elevation (m). Equation (3) gives the salt deposition index (*y*), with *T* as the topex score. A high topex score indicates a more sheltered location, so 1−*T* expresses the degree of exposure at a given site. In this model, sites that are closer to the tideline by horizontal or vertical distance will receive more deposition than sites that are farther inland and/or at higher elevations, and deposition is multiplied by the degree of topographic exposure.

### 2.6 Statistical analysis

We classified all the sample plots into vegetation groups using cluster analysis. To make the intensive plot data set parallel to the rapid assessment data, we normalized the intensive sample plot data to only include trees, shrubs, and up to three dominant herbaceous species in each plot. We then ran ordination analyses using nonmetric multidimensional scaling (NMS; Kruskal, 1964) with Sørensen distance, first on all the plots, then just on the intensively sampled plots. The cluster analysis and ordinations were performed in PC‐ORD 5.0 (McCune & Mefford, 1999).

We calculated diversity for the 400 m^2^ focal modules in the intensive plots, using the effective number of species (Hill, 1973; Jost, 2006), derived from the exponential of Shannon entropy, using Turboveg 2.1 (Hennekens & Schaminee, 2001). This measure includes relative abundance information, whereas traditional measures use only presence/absence. We calculated alpha, beta, and gamma diversity for the coastal and inland sample groups using Jost's (2006) formula to calculate effective numbers for these components of diversity. Beta diversity was calculated as the effective number of communities. We then compared species richness and effective species number of coastal and inland plots by independent samples *t* test, performed in SPSS 13 (SPSS Inc., Chicago, IL, USA).

Using data from the intensive plots, we plotted species‐area accumulation using rarefaction curves, and we extrapolated species richness‐area curves (Colwell et al., 2012) using EstimateS 9.0 (Colwell, 2013). We ran 100 randomizations for each group and extrapolated the results to sixty samples or the equivalent of a six‐hectare area.

## 3 RESULTS

### 3.1 Diversity

**Figure 3:**
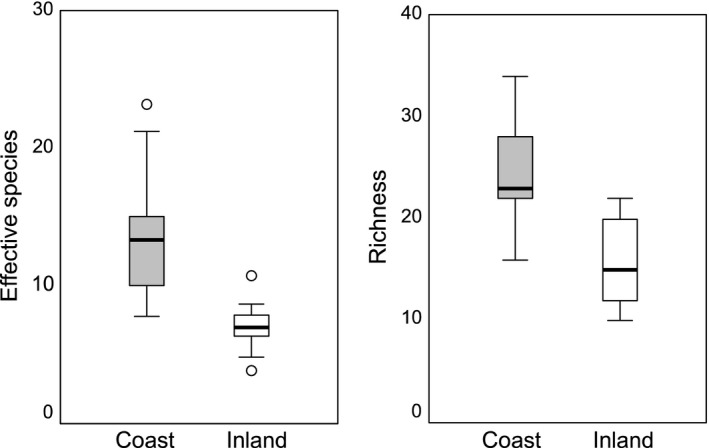
Comparison of effective species diversity and species richness between coastal and inland plots

Mean number of effective species in coastal plots was 2.1 times greater than inland plots (Figure 3). The coastal mean was 13.7, and the inland mean was 6.6 (*p *<* *.0001, *SE* 1.45). Mean species richness per 400 m^2^ in coastal plots was 1.7 times greater than inland plots. Mean coastal richness was 25, and mean inland richness was 15 (*p *<* *.0001, *SE* 1.98).

All diversity measures calculated by the exponent of Shannon entropy (Jost, 2006) were higher for coastal bluff sites than for inland sites (Table 1). Alpha diversity among coastal bluff sites was 1.9 times higher than inland sites. Beta diversity among the coastal bluff sites was 1.9 times higher than inland sites. Gamma diversity of coastal bluffs was 3.5 times higher than that of inland sites.

**Table 1:** Diversity measures for coastal and inland sites, measured by the exponential of Shannon entropy. Alpha and gamma diversity units are effective species number. Alpha diversity is per 0.1 ha. Beta diversity units are effective community numbers

Group	*n*	Alpha	Beta	Gamma
Coastal bluffs	18	13.34	5.05	67.36
Inland	9	7.02	2.72	19.11

### 3.2 Species‐area relationships

**Figure 4:**
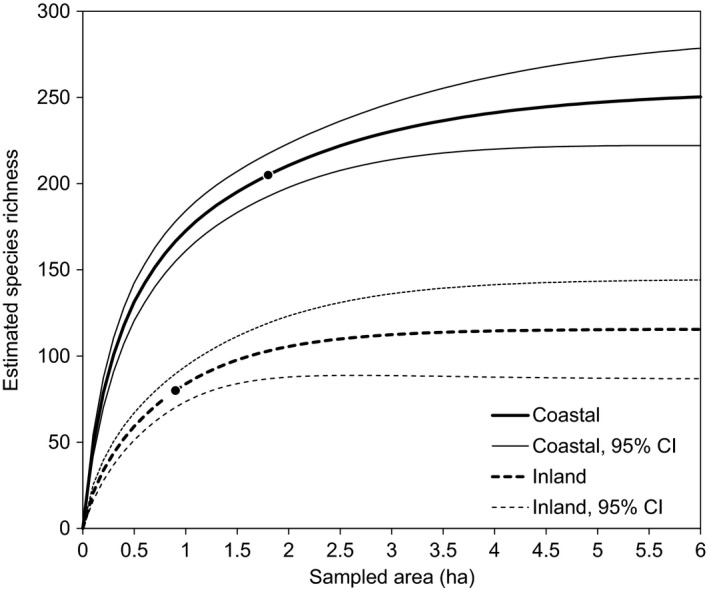
Estimated species accumulation curves for coastal bluff and inland sites. Single points indicate species richness in the total area observed (S obs), 202 spp/1.7 ha for coastal plots and 92 spp/1.0 ha for inland plots. Rarefaction curves are shown for sampled species richness up to S obs, and extrapolation curves are shown for estimated species‐area beyond S obs

Rarefaction curves for species richness of the sampled plots (S obs) and extrapolation curves for estimated species‐area beyond S obs also demonstrate higher coastal diversity (Figure 4). Smoothed species richness for the sampled coastal plots (S obs) is 173 spp. per ha, more than double that of inland plots (84 spp. per ha). The estimated richness for inland sites approaches an asymptote at 112 spp per 3 ha, whereas the estimated richness for coastal sites is again double that at 230 spp per 3 ha and does not approach an asymptote until 250 spp per 6 ha.

### 3.3 Functional groups

The cluster analysis yielded two primary groups. The larger, main group, comprising 97 plots and 190 taxa, was characterized by vegetation alliances typical of northern coastal scrub (Sawyer et al., 2009), dominated by species such as coyote brush, brambles (*Rubus parviflorus, R. spectabilis, R. ursinus*), and coffeeberry (*Frangula californica*). Plots within this main group clustered into three broad functional groups, referred to here as prairie scrub, mesic scrub, and xeric scrub, corresponding to hypothesized gradients in water availability and salt deposition. The smaller, second group consisted of 12 plots sampled from coastal bluffs, dominated by subshrubs and herbaceous perennials. We refer to this functional group as bluff scrub (Ford & Hayes, 2007; Holland, 1986). It is intermediate in composition between northern coastal scrub and herbaceous dune mat vegetation of the coastal strand.

### 3.4 Ordination

**Figure 5:**
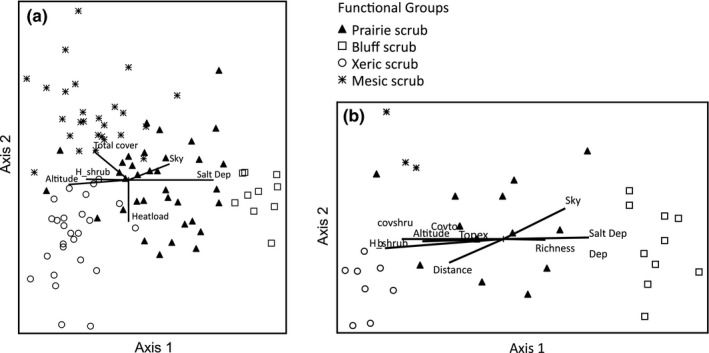
(a) Nonmetric multidimensional scaling (NMS) ordination of all intensive and rapid assessment plots, showing functional groups with joint plot of environmental correlates. (b) NMS ordination of just the intensive plots, rotated by species richness. Environmental factors are labeled as follows: H_shrub = mean shrub height, Sky = cloud frequency, Heatload = heat load index, Salt_Dep = salt deposition index, Total cover & Covtot = total vegetative cover, covshru = shrub cover, Distance = distance to coast. The plots are divided into four functional groups: prairie scrub, bluff scrub, xeric scrub, and mesic scrub

Indirect gradient analysis of all intensive and rapid assessment plots, using NMS ordination, indicated a three‐axis solution was best (Monte Carlo *p* = .0196, Axis 1 *R* ^2^ = .392, Axis 2 *R* ^2^ = .292, Axis 3 *R* ^2^ = .129, Axis 1 & 2 *R* ^2^ = .684, total *R* ^2^ = .813). The ordination yielded four functional groups on two axes, with a joint plot overlay of the best correlated environmental variables (Pearson's *r *>* *.26; Figure 5a). Axis 1 is interpreted as a salt deposition gradient. The salt deposition index (Salt_Dep) was the environmental variable most strongly correlated with variation on Axis 1 (Pearson's *r *=* *.736). Elevation was inversely correlated with salt deposition along Axis 1 (*r *=* *−.625), as predicted by the salt deposition model. Shrub height (H_shrub) showed an inverse correlation with salt deposition along this axis (*r *=* *−.520), suggesting that vegetation height is suppressed by salt deposition. Percent cloud cover (Sky) was also correlated with Axis 1 (*r *=* *.522). The functional groups were organized along Axis 1 as follows: bluff scrub, prairie scrub, mesic scrub, xeric scrub. Axis 2 is interpreted as vegetation response along a gradient of water availability. Heat load (Heatload) was the environmental variable most strongly correlated with variation on Axis 2 (*r *=* *−.514). The xeric coastal scrub functional group showed a strong negative correlation with Axis 2, while the mesic coastal scrub showed a strong positive correlation with Axis 2.

Nonmetric multidimensional scaling ordination of only the intensive sample plots, rotated on Axis 1 by species richness, showed a positive relationship between species richness (Richness) and the index of salt deposition (Salt_Dep) and cloud frequency (Sky; Figure 5b). Shrub height (H_shrub), shrub cover (covshrub), elevation, total vegetative cover (Covtot), and topographic exposure (Topex) were also correlated. We interpret Axis 1 as a gradient of salt deposition (salt deposition index, Pearson's *r* = .698). Species richness was positively correlated with Axis 1 (Pearson's *r* = .484).

### 3.5 Soil characteristics

No significant correlations were found between either soil organic matter or mineral nutrients and vegetation pattern or diversity (results not shown).

## 4 DISCUSSION

Vascular plant diversity increases significantly with maritime influence in coastal shrublands of Central California, paralleling previous work showing a general coast–inland biodiversity gradient in other vegetation types in this region (Harrison et al., 2008; Stromberg et al., 2001; Vasey, Loik, & Parker, 2012). Combined, these studies suggest where intensive conservation efforts should focus. Endemic species richness is concentrated in coastal areas, particularly in the San Francisco Bay Area (Ackerly, 2009; Kraft et al., 2010), and is especially concentrated along the immediate coast. Within northern coastal scrub, plant diversity and species richness were consistently higher in coastal plots than in inland plots. Diversity in coastal plots was 2.1 times higher than inland plots, and species richness was 1.7 times higher (Figure 3). Beta diversity among the coastal bluff sites also was nearly twice as high as inland sites, with 5.0 equally likely, distinct communities at the coast compared to 2.72 inland (Table 1). Estimated richness by rarefaction was far greater for coastal bluff sites (230 spp/3 ha) than for inland sites (112 spp/3 ha; Figure 4).

Local diversity in coastal scrub correlated the most with aerosol salt deposition and with processes that moderate the Mediterranean‐climate summer rainless period. Salt deposition was the abiotic process most strongly correlated with plant diversity and vegetation composition. Next to coastal cliffs, salt deposition opens shrub canopies, permitting a diverse array of herbaceous species occupying the disturbance gaps (Baxter & Parker, 1999). In this study, herbaceous cover and species richness both increased with increased salt deposition, as measured by the salt deposition index, while shrub height and cover decreased (Figure 5b). The salt deposition index showed a much stronger signal than topex alone, and local wind speed showed no correlation. While wind exposure can be a significant local influence, in these coastal sites, wind exposure in itself did not explain as much variance in herbaceous cover and species richness as salt deposition. At the high end of the salt deposition index in the ordination, the herbaceous vegetation layer was dominant, with woody plants present only in dwarfed forms and low cover, as in the bluff scrub community.

Salt exposure on coastal bluffs represents a relatively constant disturbance regime of fluctuating intensity. Maritime salt spray has been shown to enter coastal plants primarily through foliar absorption. This causes leaf necrosis when sodium chloride ions penetrate through small lacerations in the cuticle caused by wind buffeting, leading to ion toxicity and loss of photosynthetic tissue. Salt spray has been shown to cause far greater damage to plant tissues in dune systems than wind desiccation alone (Boyce, 1954; Clayton, 1972; Griffiths & Orians, 2003). The background level of salt exposure on coastal bluffs is punctuated regularly by periods of heavy surf and high exposure to aerosols. To maintain viable populations in this environment, plant species must possess adaptations for salt tolerance. Shrub species with high intrinsic reproduction, high growth rates, and salt‐tolerant tissues such as coyote brush and lizard tail (*Eriophyllum staechadifolium*) are capable of colonizing and dominating small patches by either priority effects or competition. However, as these plants grow taller, their meristems become more exposed to airborne salts. Punctuated winter storm events deposit large quantities of salts on coastal bluffs from wave activity and can cause extensive foliage dieback in coastal vegetation (personal observation). Frequent salt‐induced disturbance in the most exposed coastal sites may maintain high plant diversity (Baxter & Parker, 1999) by keeping the community far from competitive equilibrium (Huston, 1979; Huston & Huston, 1994). It is well established that salt deposition is a major disturbance factor structuring coastal strand vegetation at the immediate coastline (Barbour, 1978; Barbour & DeJong, 1977; Boyce, 1954). This study indicates that salt exposure is also an important factor in upland vegetation dynamics of coastal California, which must be considered along with well‐known MTC ecosystem disturbance factors such as fire and grazing (Cowling, Rundel, Lamont, Arroyo, & Arianoutsou, 1996; Grace & Keeley, 2006; Harrison et al., 2003; Naveh & Whittaker, 1980). Moreover, salt spray is a naturally generated disturbance factor, while fire and grazing are largely human‐caused disturbances in California's coastal zone. Fire ignitions in coastal California are predominantly anthropogenic due to the rarity of lightning‐ignited fires in this area (Stephens, Martin, & Clinton, 2007). High intensity livestock grazing was introduced across California during the European settlement period and continues to this day. There had not been such persistent grazing prior to this since a megafaunal extinction event over 10,000 years ago (Edwards, 2007).

Water availability is also a major environmental factor influencing vegetation patterns in NCS, as inferred by alignment of the mesic and xeric scrub groups along NMS axes, correlation with the heat load index, and other well‐established proxy metrics (Figure 5a). Species with high fidelity to the xeric coastal scrub functional group (e.g., *Artemisia californica*,* Mimulus aurantiacus*,* Stipa lepida*) are known to be associated with drier environments in contrast to species with high fidelity to the mesic coastal scrub functional group (e.g., *Rubus ursinus*,* Corylus cornuta*,* Polystichum munitum*) that are associates of moister environments (Baldwin et al., 2012). Leaf size, specific leaf area, wood density, and maximum height also covary strongly with species distributions along a soil moisture availability gradient in woody plant communities of coastal California (Ackerly & Cornwell, 2007; Ackerly, Knight, Weiss, Barton, & Starmer, 2002; Cornwell & Ackerly, 2009). Mean leaf size of woody species reported by Ackerly and Cornwell (2007) was well correlated with NMS Axis 2 in our results. Larger‐leaved species were observed more often in the mesic scrub group, and smaller‐leaved species were observed more often in the xeric scrub group. Additionally, elevation, slope, and aspect patterns in the data support the interpretation of water availability being a significant process (Cornwell & Ackerly, 2009; Franklin, 1998; Keeley & Keeley, 1988; Moody & Meentemeyer, 2001; Poole & Miller, 1981; Westman, 1981), as well as heat load (McCune & Keon, 2002), and thus potential evapotranspiration (PET) among sites at a fine scale. As a whole, in ordinations, mesic scrub plots were in opposition to plots in the xeric scrub group (Figure 5a,b).

The diversity patterns described in this study suggest disturbance‐productivity interactions (Huston, 1979, 2014; Kondoh, 2001). Higher water availability at the coast in the summer and more frost‐free days in the wet winter (NOAA, 2013) mean that annual productivity will generally be higher in coastal zones than inland zones. Marine fog and stratus reduce PET by cloud shading and add significant summer precipitation from fog drip at the elevation of the cloud ceiling (Fischer et al., 2009). Higher species diversity was observed in sites with relatively high estimated productivity (water availability) and high disturbance (salt exposure). Disturbances that open shrub canopies dominated by superior competitors should increase species richness by freeing resources for recruitment in the herbaceous layer, which typically supports greater species richness at multiple scales. In this model, patches with higher soil moisture during the growing season, thus higher productivity, will support higher herbaceous plant diversity. Conversely, lower species diversity was observed in drier sites with lower estimated productivity and disturbance.

## 5 CONCLUSION

A general phenomenon of increasing diversity with maritime influence appears to occur in all vegetation types investigated in central California. Northern coastal scrub exhibits marked gradients of plant diversity and strong differences between coastal and inland stands. These diversity gradients were correlated with maritime influence and were significant at local to regional scales in this study. The maritime influence is likely principally driven by two abiotic processes, salt exposure, and water availability. Both processes are highest near the coast and decline inland. Within local sites, diversity varied as mosaics created by local topography influencing wind, salt exposure, heat load, and water availability.

These results emphasize the importance of plant conservation in California's coastal zone, especially in unprotected areas, which tend to host high concentrations of rare and endemic taxa (Kraft et al., 2010; Pavlik & Skinner, 1994). Coastal bluffs and terraces along the coast of California have high conservation value, as these areas have been heavily impacted by development and agriculture. The coastal climate and heterogeneous topography is also likely to provide habitat refugia from temperature extremes associated with climate change. The salt deposition index presented here, fog and low cloud indices (Torregrosa, Combs, & Peters, 2016), and water availability models such as Basin Characterization (Flint, Flint, Thorne, & Boynton, 2013), can be used in overlay analyses to predict mesoscale diversity hotspots, which may be followed by botanical inventories to prioritize protection. To improve the conservation status of NCS on protected lands, threat mitigations may include reduction in anthropogenic disturbance (e.g., overgrazing, off‐road vehicle use), erosion control, and control of nonindigenous plant invasions. Introducing intermediate levels of disturbance such as shrub removal, limited grazing, or burning may increase plant diversity on relatively productive sites where shrub expansion is reducing plant diversity, but the effects of these types of disturbance in MTC shrublands and grasslands are highly variable and contingent on local conditions (Grace & Keeley, 2006; Harrison et al., 2003) and should be approached experimentally. Finally, restoration of degraded sites must remediate soil disturbance, restore hydrologic function, and revegetate with plant taxa from nearby reference sites with similar topographic exposure. These recommendations are likely to apply to coastal zones elsewhere, where there are steep maritime to continental climatic gradients, especially in other MTC regions where maritime influences may reduce summer moisture stress.

## CONFLICT OF INTEREST

None declared.

## AUTHOR CONTRIBUTIONS

V. Thomas Parker and Eric Wrubel generated the hypothesis and designed the study; Eric Wrubel collected the data and conducted statistical and spatial analyses; Eric Wrubel and V. Thomas Parker wrote the manuscript.

## References

[ece34104-bib-0001] Ackerly, D. D. (2009). Evolution, origin and age of lineages in the Californian and Mediterranean floras. Journal of Biogeography, 36(7), 1221–1233. 10.1111/j.1365-2699.2009.02097.x

[ece34104-bib-0002] Ackerly, D. D. , & Cornwell, W. K. (2007). A trait‐based approach to community assembly: Partitioning of species trait values into within‐and among‐community components. Ecology Letters, 10(2), 135–145. 10.1111/j.1461-0248.2006.01006.x 17257101

[ece34104-bib-0003] Ackerly, D. D. , Knight, C. , Weiss, S. , Barton, K. , & Starmer, K. (2002). Leaf size, specific leaf area and microhabitat distribution of chaparral woody plants: Contrasting patterns in species level and community level analyses. Oecologia, 130(3), 449–457. 10.1007/s004420100805 28547053

[ece34104-bib-0004] Ackerly, D. D. , Loarie, S. R. , Cornwell, W. K. , Weiss, S. B. , Hamilton, H. , Branciforte, R. , & Kraft, N. J. B. (2010). The geography of climate change: Implications for conservation biogeography. Diversity and Distributions, 16, 476–487. 10.1111/j.1472-4642.2010.00654.x

[ece34104-bib-0005] Baldwin B. G., Goldman D. H., Keil D. J., Patterson R., Rosatti T. J., & Wilken D. H. (Eds.) (2012). The Jepson manual: Vascular plants of California (2nd ed.). Berkeley and Los Angeles, CA: University of California Press.

[ece34104-bib-0006] Barbour, M. G. (1978). Salt spray as a microenvironmental factor in the distribution of beach plants at Point Reyes, California. Oecologia, 32(2), 213–224. 10.1007/BF00366073 28309399

[ece34104-bib-0007] Barbour, M. G. , & DeJong, T. M. (1977). Response of west coast beach taxa to salt spray, seawater inundation, and soil salinity. Bulletin of the Torrey Botanical Club, 104, 29–34. 10.2307/2484662

[ece34104-bib-0008] Baxter, J. W. , & Parker, V. T. (1999). Canopy gaps, zonation and topography structure: A northern coastal scrub community on California coastal bluffs. Madroño, 46(2), 69–79.

[ece34104-bib-0009] Blanchard, D. C. , & Woodcock, A. H. (1980). The production, concentration, and vertical distribution of the sea‐salt aerosol. Annals of the New York Academy of Sciences, 338(1), 330–347. 10.1111/j.1749-6632.1980.tb17130.x

[ece34104-bib-0010] Boyce, S. G. (1954). The salt spray community. Ecological Monographs, 24(1), 29–67. 10.2307/1943510

[ece34104-bib-0011] Brewer, R. , & McCann, M. T. (1982). Laboratory and field methods in ecology. Philadelphia, PA: Saunders College Publishing.

[ece34104-bib-0012] Brooks, T. M. , Mittermeier, R. A. , da Fonseca, G. A. , Gerlach, J. , Hoffmann, M. , Lamoreux, J. F. , … Rodrigues, A. S. (2006). Global biodiversity conservation priorities. Science, 313(5783), 58–61. 10.1126/science.1127609 16825561

[ece34104-bib-0013] Burge, D. O. , Thorne, J. H. , Harrison, S. P. , O'Brien, B. C. , Rebman, J. P. , Shevock, J. R. , … Oberbauer, T. A. (2016). Plant diversity and endemism in the California Floristic Province. Madroño, 63(2), 3–206.

[ece34104-bib-0014] Calflora (2015). Information on California plants for education, research and conservation [web application]. Berkeley, CA: The Calflora Database. Retrieved from http://www.calflora.org.

[ece34104-bib-0015] Casazza, G. , Zappa, E. , Mariotti, M. G. , Médail, F. , & Minuto, L. (2008). Ecological and historical factors affecting distribution pattern and richness of endemic plant species: The case of the Maritime and Ligurian Alps hotspot. Diversity and Distributions, 14(1), 47–58. 10.1111/j.1472-4642.2007.00412.x

[ece34104-bib-0016] Consortium of California Herbaria (CCH ) (2015). Consortium of California Herbaria [Data file]. Retrieved from http://ucjeps.berkeley.edu/consortium/

[ece34104-bib-0017] Clayton, J. L. (1972). Salt spray and mineral cycling in two California coastal ecosystems. Ecology, 53(1), 74–81. 10.2307/1935711

[ece34104-bib-0018] California Native Plant Society (CNPS ) (2007). Vegetation rapid assessment protocol. California Native Plant Society Vegetation Committee. Retrieved from http://www.cnps.org/cnps/vegetation/pdf/protocol-combined.pdf

[ece34104-bib-0019] California Native Plant Society (CNPS ) (2015). Inventory of rare and endangered plants of California [online edition, v8‐03 0.39]. California Native Plant Society, Rare Plant Program. Retrieved from http://www.rareplants.cnps.org

[ece34104-bib-0020] Colwell, R. K. (2013). EstimateS: Statistical estimation of species richness and shared species from samples. Version 9. Retrieved from http://purl.oclc.org/estimates

[ece34104-bib-0021] Colwell, R. K. , Chao, A. , Gotelli, N. J. , Lin, S. Y. , Mao, C. X. , Chazdon, R. L. , & Longino, J. T. (2012). Models and estimators linking individual‐based and sample‐based rarefaction, extrapolation and comparison of assemblages. Journal of Plant Ecology, 5(1), 3–21. 10.1093/jpe/rtr044

[ece34104-bib-0022] Cornwell, W. K. , & Ackerly, D. D. (2009). Community assembly and shifts in plant trait distributions across an environmental gradient in coastal California. Ecological Monographs, 79(1), 109–126. 10.1890/07-1134.1

[ece34104-bib-0023] Cowling, R. M. , & Lombard, A. T. (2002). Heterogeneity, speciation/extinction history and climate: Explaining regional plant diversity patterns in the Cape Floristic Region. Diversity and Distributions, 8(3), 163–179. 10.1046/j.1472-4642.2002.00143.x

[ece34104-bib-0024] Cowling, R. M. , Rundel, P. W. , Lamont, B. B. , Arroyo, M. K. , & Arianoutsou, M. (1996). Plant diversity in Mediterranean‐climate regions. Trends in Ecology & Evolution, 11(9), 362–366. 10.1016/0169-5347(96)10044-6 21237880

[ece34104-bib-0025] Daubenmire, R. F. (1968). Plant communities: A textbook of plant synecology. New York, NY: Harper & Row.

[ece34104-bib-0026] Dukes, J. S. , & Mooney, H. A. (2004). Disruption of ecosystem processes in western North America by invasive species. Revista Chilena de Historia Natural, 77(3), 411–437.

[ece34104-bib-0027] Edwards, S. W. (2007). Rancholabrean mammals of California and their relevance for understanding modern plant ecology In Stromberg M. R., Corbin J. D., & D'Antonio C. M. (Eds.), California Grasslands (pp. 48–52). Berkeley and Los Angeles, CA: University of California Press.

[ece34104-bib-0028] Ferrier, S. , & Drielsma, M. (2010). Synthesis of pattern and process in biodiversity conservation assessment: A flexible whole‐landscape modelling framework. Diversity and Distributions, 16(3), 386–402. 10.1111/j.1472-4642.2010.00657.x

[ece34104-bib-0029] Fischer, D. T. , Still, C. J. , & Williams, A. P. (2009). Significance of summer fog and overcast for drought stress and ecological functioning of coastal California endemic plant species. Journal of Biogeography, 36(4), 783–799. 10.1111/j.1365-2699.2008.02025.x

[ece34104-bib-0030] Flint, L. E. , Flint, A. L. , Thorne, J. H. , & Boynton, R. (2013). Fine‐scale hydrologic modeling for regional landscape applications: The California Basin Characterization Model development and performance. Ecological Processes, 2(1), 25 10.1186/2192-1709-2-25

[ece34104-bib-0031] Ford, L. D. , & Hayes, G. F. (2007). Northern coastal scrub and coastal prairie In Barbour M., Keeler‐Wolf T., & Shoenherr A. A. (Eds.), Terrestrial vegetation of California (3rd ed., pp. 180–207). Berkeley, CA: University of California Press 10.1525/california/9780520249554.001.0001

[ece34104-bib-0032] Franklin, J. (1998). Predicting the distribution of shrub species in southern California from climate and terrain‐derived variables. Journal of Vegetation Science, 9, 733–748. 10.2307/3237291

[ece34104-bib-0033] Gaston, K. J. (2000). Global patterns in biodiversity. Nature, 405(6783), 220–227. 10.1038/35012228 10821282

[ece34104-bib-0034] Gillison, A. N. , & Brewer, K. R. W. (1985). The use of gradient directed transects or gradsects in natural resource surveys. Journal of Environmental Management, 20, 103–127.

[ece34104-bib-0035] Grace, J. B. , & Keeley, J. E. (2006). A structural equation model analysis of postfire plant diversity in California shrublands. Ecological Applications, 16(2), 503–514. 10.1890/1051-0761(2006)016[0503:ASEMAO]2.0.CO;2 16711040

[ece34104-bib-0036] Griffiths, M. E. , & Orians, C. M. (2003). Salt spray differentially affects water status, necrosis, and growth in coastal sandplain heathland species. American Journal of Botany, 90(8), 1188–1196. 10.3732/ajb.90.8.1188 21659219

[ece34104-bib-0037] Harrison, S. , Inouye, B. D. , & Safford, H. D. (2003). Ecological heterogeneity in the effects of grazing and fire on grassland diversity. Conservation Biology, 17(3), 837–845. 10.1046/j.1523-1739.2003.01633.x

[ece34104-bib-0038] Harrison, S. , Viers, J. H. , & Quinn, J. F. (2008). Climatic and spatial patterns of diversity in the serpentine plants of California. Diversity and Distributions, 6(3), 153–162.

[ece34104-bib-0039] Hennekens, S. M. , & Schaminee, J. H. J. (2001). Turboveg, a comprehensive database management system for vegetation data. Journal of Vegetation Science, 12, 589–591. 10.2307/3237010

[ece34104-bib-0040] Hijmans, R. J. , Cameron, S. E. , Parra, J. L. , Jones, P. G. , & Jarvis, A. (2005). Very high resolution interpolated climate surfaces for global land areas. International Journal of Climatology, 25(15), 1965–1978. 10.1002/(ISSN)1097-0088

[ece34104-bib-0041] Hill, M. O. (1973). Diversity and evenness: A unifying notation and its consequences. Ecology, 54(2), 427–432. 10.2307/1934352

[ece34104-bib-0042] Holland, R. (1986). Preliminary descriptions of the terrestrial natural communities of California. Sacramento, CA: Unpublished document, California Department of Fish and Game, Natural Heritage Division.

[ece34104-bib-0043] Huston, M. A. (1979). A general hypothesis of species diversity. The American Naturalist, 113(1), 81–101. 10.1086/283366

[ece34104-bib-0044] Huston, M. A. (2014). Disturbance, productivity, and species diversity: Empiricism vs. logic in ecological theory. Ecology, 95(9), 2382–2396. 10.1890/13-1397.1

[ece34104-bib-0045] Huston, M. A. , & Huston, M. A. (1994). Biological diversity: The coexistence of species. Cambridge, UK: Cambridge University Press.

[ece34104-bib-0046] Jost, L. (2006). Entropy and diversity. Oikos, 113, 363–375. 10.1111/j.2006.0030-1299.14714.x

[ece34104-bib-0047] Keeley, J. E. , & Keeley, S. C. (1988). Chaparral In Barbour M. G. & Billings W. D. (Eds.), North American terrestrial vegetation (pp. 165–207). Cambridge, UK: Cambridge University Press.

[ece34104-bib-0048] Keeley, J. E. , & Swift, C. C. (1995). Biodiversity and ecosystem functioning in Mediterranean‐climate California In Mediterranean‐type ecosystems (pp. 121–183). Berlin and Heidelberg, Germany: Springer 10.1007/978-3-642-78881-9

[ece34104-bib-0049] Kondoh, M. (2001). Unifying the relationships of species richness to productivity and disturbance. Proceedings of the Royal Society of London B: Biological Sciences, 268(1464), 269–271. 10.1098/rspb.2000.1384 PMC1088602 11217897

[ece34104-bib-0050] Kraft, N. J. , Baldwin, B. G. , & Ackerly, D. D. (2010). Range size, taxon age and hotspots of neoendemism in the California flora. Diversity and Distributions, 16(3), 403–413. 10.1111/j.1472-4642.2010.00640.x

[ece34104-bib-0051] Kremen, C. , Cameron, A. , Moilanen, A. , Phillips, S. J. , Thomas, C. D. , Beentje, H. , … Harper, G. J. (2008). Aligning conservation priorities across taxa in Madagascar with high‐resolution planning tools. Science, 320(5873), 222–226. 10.1126/science.1155193 18403708

[ece34104-bib-0052] Kruskal, J. B. (1964). Multidimensional scaling by optimizing goodness of fit to a nonmetric hypothesis. Psychometrika, 29(1), 1–27. 10.1007/BF02289565

[ece34104-bib-0053] Linder, H. P. (1991). Environmental correlates of patterns of species richness in the south‐western Cape Province of South Africa. Journal of Biogeography, 18, 509–518. 10.2307/2845687

[ece34104-bib-0054] McCune, B. , & Keon, D. (2002). Equations for potential annual direct incident radiation and heat load. Journal of Vegetation Science, 13(4), 603–606. 10.1111/j.1654-1103.2002.tb02087.x

[ece34104-bib-0055] McCune, B. , & Mefford, M. J. (1999). PC‐ord. Multivariate analysis of ecological data, version, 4(0).

[ece34104-bib-0056] Meira, G. R. , Andrade, M. C. , Alonso, C. , Padaratz, I. J. , & Borba, J. C. (2007). Salinity of marine aerosols in a Brazilian coastal area—Influence of wind regime. Atmospheric Environment, 41(38), 8431–8441. 10.1016/j.atmosenv.2007.07.004

[ece34104-bib-0057] Meira, G. R. , Andrade, M. C. , Padaratz, I. J. , Alonso, M. C. , & Borba, J. C. (2006). Measurements and modelling of marine salt transportation and deposition in a tropical region in Brazil. Atmospheric Environment, 40(29), 5596–5607. 10.1016/j.atmosenv.2006.04.053

[ece34104-bib-0058] Mittermeier, R. A. , Turner, W. R. , Larsen, F. W. , Brooks, T. M. , & Gascon, C. (2011). Global biodiversity conservation: The critical role of hotspots In Zachos F. E. & Habel J. C. (Eds.), Biodiversity hotspots (pp. 3–22). Berlin and Heidelberg, Germany: Springer 10.1007/978-3-642-20992-5

[ece34104-bib-0059] Moody, A. , & Meentemeyer, R. K. (2001). Environmental factors influencing spatial patterns of shrub diversity in chaparral, Santa Ynez Mountains, California. Journal of Vegetation Science, 12(1), 41–52. 10.1111/j.1654-1103.2001.tb02615.x

[ece34104-bib-0060] Munz, P. A. , & Keck, D. D. (1959). A California flora. Berkeley, CA: University of California Press.

[ece34104-bib-0061] Myers, N. , Mittermeier, R. A. , Mittermeier, C. G. , da Fonseca, G. A. , & Kent, J. (2000). Biodiversity hotspots for conservation priorities. Nature, 403(6772), 853–858. 10.1038/35002501 10706275

[ece34104-bib-0062] Naveh, Z. , & Whittaker, R. H. (1980). Structural and floristic diversity of shrublands and woodlands in northern Israel and other Mediterranean areas. Vegetatio, 41(3), 171–190. 10.1007/BF00052445

[ece34104-bib-0063] NOAA (2013), National Climatic Data Center [Data file]. National Oceanic and Atmospheric Administration. Retrieved from: http://www.ncdc.noaa.gov/climate-information

[ece34104-bib-0064] NREL (2003). “ca_50mwind”. vector digital data [Data file]. National Renewable Energy Laboratory. Retrieved from: http://www.nrel.gov/gis/cfm/data/GIS_Data_Technology_Specific/United_States/Wind/High_Resolution/California_Wind_High_Resolution.zip

[ece34104-bib-0065] Parker, V. T. , Schile, L. M. , Vasey, M. C. , & Callaway, J. C. (2011). Efficiency in assessment and monitoring methods: Scaling down gradient‐directed transects. Ecosphere, 2(9), 1–11.

[ece34104-bib-0066] Pavlik, B. M. , & Skinner, M. W. (1994). Ecological characteristics of California's rare plants In Skinner M. W., & Pavlik B. M. (Eds.), Inventory of rare and endangered vascular plants of California [Special Publication No. 1, 5th ed.] (pp. 4–6). Sacramento, CA: California Native Plant Society.

[ece34104-bib-0067] Poole, D. K. , & Miller, P. C. (1981). The distribution of plant water stress and vegetation characteristics in southern California chaparral. American Midland Naturalist, 105, 32–43. 10.2307/2425007

[ece34104-bib-0068] Quine, C. P. , & White, I. M. S. (1994). Using the relationship between rate of tatter and topographic variables to predict site windiness in upland Britain. Forestry, 67(3), 245–256. 10.1093/forestry/67.3.245

[ece34104-bib-0069] Raven, P. H. , & Axelrod, D. I. (1978). Origin and relationships of the California flora. Berkeley, CA: University of California Press.

[ece34104-bib-0070] Richerson, P. J. , & Lum, L. K. (1980). Patterns of plant species diversity in California: Relation to weather and topography. American Naturalist, 116, 504–536. 10.1086/283645

[ece34104-bib-0071] Rundel, P. W. , Arroyo, M. T. , Cowling, R. M. , Keeley, J. E. , Lamont, B. B. , & Vargas, P. (2016). Mediterranean biomes: Evolution of their vegetation, floras, and climate. Annual Review of Ecology, Evolution, and Systematics, 47, 383–407. 10.1146/annurev-ecolsys-121415-032330

[ece34104-bib-0072] Sawyer, J. O. , Keeler‐Wolf, T. , & Evens, J. (2009). A manual of California vegetation. Sacramento, CA: California Native Plant Society Press.

[ece34104-bib-0073] Stebbins, G. L. , & Major, J. (1965). Endemism and speciation in the California flora. Ecological Monographs, 35(1), 2–35.

[ece34104-bib-0074] Stephens, S. L. , Martin, R. E. , & Clinton, N. E. (2007). Prehistoric fire area and emissions from California's forests, woodlands, shrublands, and grasslands. Forest Ecology and Management, 251(3), 205–216. 10.1016/j.foreco.2007.06.005

[ece34104-bib-0075] Stromberg, M. R. , Kephart, P. , & Yadon, V. (2001). Composition, invasibility, and diversity in coastal California grasslands. Madroño, 48(4), 236–252.

[ece34104-bib-0076] Tang, Z. (2008). Evaluating local coastal zone land use planning capacities in California. Ocean & Coastal Management, 51(7), 544–555. 10.1016/j.ocecoaman.2008.06.001

[ece34104-bib-0077] Torregrosa, A. , Combs, C. , & Peters, J. (2016). GOES‐derived fog and low cloud indices for coastal north and central California ecological analyses. Earth and Space Science, 3, 46–67. 10.1002/2015EA000119

[ece34104-bib-0078] USDA (2003). Forest Service, Pacific Southwest Region, Remote Sensing Lab. *Existing Vegetation – CALVEG, Eveg tiles 22A, 22B, 24, 25, 26* [Data file].

[ece34104-bib-0079] USGS (2012). National elevation dataset. U.S. Geological Survey, Sioux Falls, SD.

[ece34104-bib-0080] Vasey, M. C. , Loik, M. E. , & Parker, V. T. (2012). Influence of summer marine fog and low cloud stratus on water relations of evergreen woody shrubs (*Arctostaphylos*: Ericaceae) in the chaparral of central California. Oecologia, 170(2), 325–337. 10.1007/s00442-012-2321-0 22526938

[ece34104-bib-0081] Vasey, M. C. , Parker, V. T. , Holl, K. D. , Loik, M. E. , & Hiatt, S. (2014). Maritime climate influence on chaparral composition and diversity in the coast range of central California. Ecology and Evolution, 4(18), 3662–3674. 10.1002/ece3.1211 25478156 PMC4224539

[ece34104-bib-0082] Westman, W. E. (1981). Factors influencing the distribution of species of Californian coastal sage scrub. Ecology, 62, 439–455. 10.2307/1936717

[ece34104-bib-0083] Williams, K. J. , Ford, A. , Rosauer, D. F. , De Silva, N. , Mittermeier, R. , Bruce, C. , … Margules, C. (2011). Forests of East Australia: The 35th biodiversity hotspot In Zachos F. E., & Habel J. C. (Eds.), Biodiversity hotspots (pp. 295–310). Berlin and Heidelberg, Germany: Springer 10.1007/978-3-642-20992-5

[ece34104-bib-0084] Wilson, J. D. (1984). Determining a topex score. Scottish Forestry, 38(4), 251–256.

