# Supplementary figures and images for: Local patterns of diversity in California northern coastal scrub
Source: Ecol Evol. 2018 Jun 27;8(15):7250–60. doi: 10.1002/ece3.4104 (PMC6106371; doi:10.1002/ece3.4104)

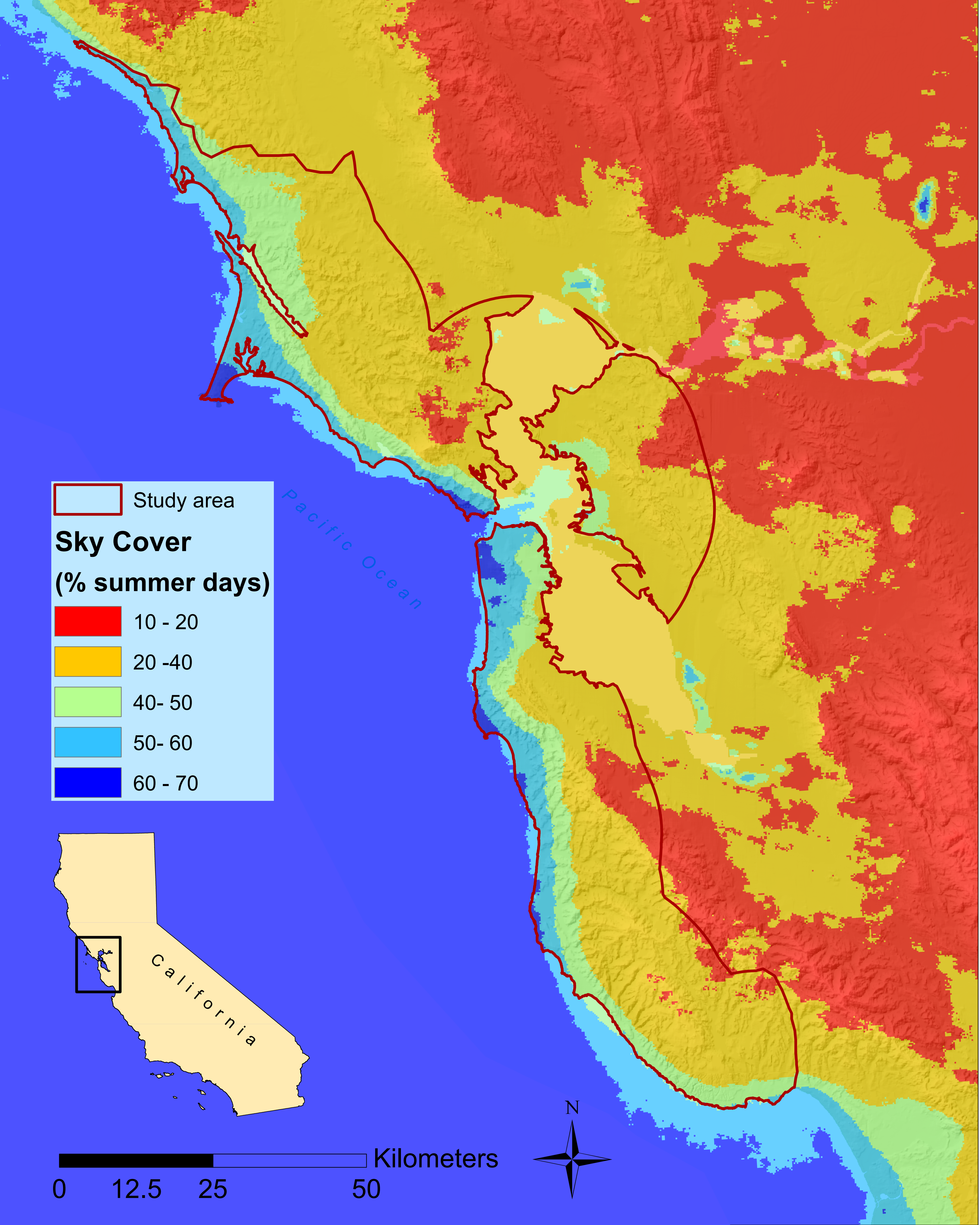

Study area

**Sky Cover**  
**(% summer days)**

|  |         |
|--|---------|
|  | 10 - 20 |
|  | 20 - 40 |
|  | 40 - 50 |
|  | 50 - 60 |
|  | 60 - 70 |

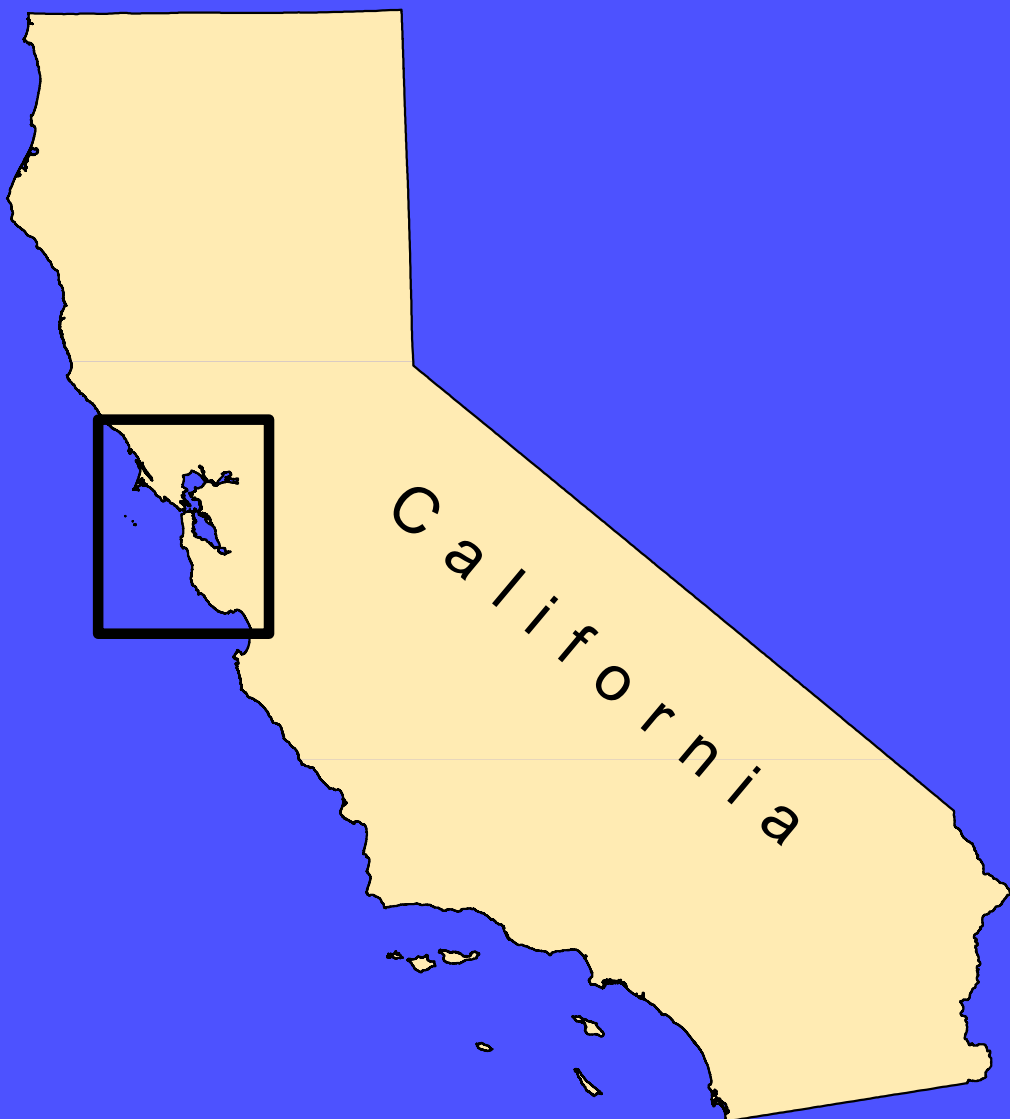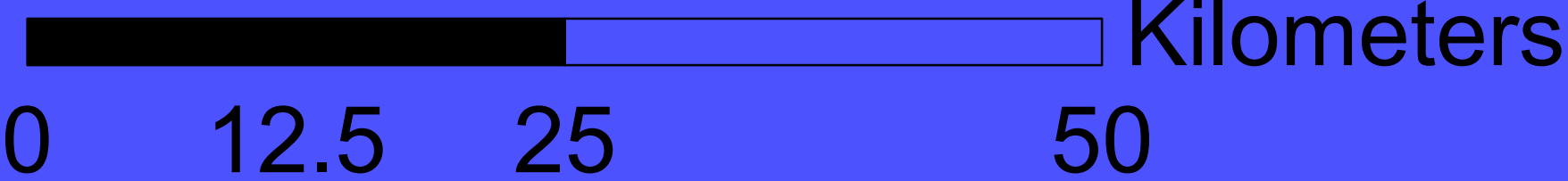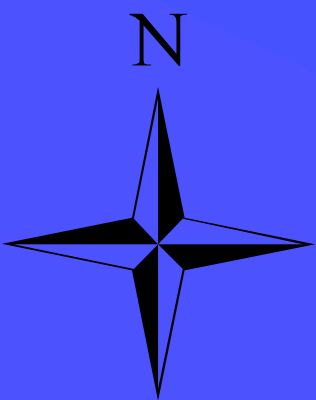

Supplement: Supplementary file 1 [file ECE3-8-7250-s001.pdf]
