# Supplementary material for: Local patterns of diversity in California northern coastal scrub
Source: Ecol Evol. 2018 Jun 27;8(15):7250–60. doi: 10.1002/ece3.4104 (PMC6106371; doi:10.1002/ece3.4104)
Supplement: Supplementary file 6 [file ECE3-8-7250-s006.pdf]

Master vascular plant list<br>Local patterns of diversity in California northern coastal scrub<br>E. Wrubel, V.T Parker	
**Scientific Name**	**Common Name**
Acaena pinnatifida Ruiz & Pav.	Argentinian biddy-biddy
Achillea millefolium L.	common 1arrow
Agoseris apargioides (Less.) Greene var. eastwoodiae (Fedde) Munz	woolly goat chicory
Agrosits densiflora Vasey	California bentgrass
Agrostis pallens Trin	seashore bentgrass
Aira caryophyllea L.	silver hairgrass
Aira praecox L.	yellow hairgrass
Allium dichlamydeum Greene	coastal onion
Ambrosia chamissonis (Less.) E. Greene	silver beachweed
Amsinckia spectabilis Fischer & C. Meyer	seaside fiddleneck
Anagallis arvensis L.	scarlet pimpernel
Anaphalis margaritacea (L.) Benth.	western pearly everlasting
Angelica hendersonii J.M. Coult. & Rose	Henderson's angelica
Anthriscus caucalis M. Bieb.	bur chervil
Apiastrum angustifolium Nutt.	mock parsley
Arabis blepharophylla Hook. & Arn.	coast rockcress
Armeria maritima (Mill.) Willd. var. californica (Boiss.) G.H.M. Lawrence	California sea pink
Artemisia californica Less.	coastal sagebrush
Artemisia douglasiana Besser	mugwort
Artemisia pycnocephala (Less.) DC.	beach wormwood
Symphiotrichium chilense (Nees) G.L. Nesom	Pacific aster
Astragalus nuttallii (Torr. & A. Gray) J.T. Howell var. nuttallii	Nuttall's milkvetch
Astragalus pycnostachyus A. Gray var. pycnostachyus	marsh milkvetch
Avena barbata Pott ex Link	slender oat
Baccharis pilularis DC.	coyotebrush
Berberis pinnata Lagasca	California barberry
Bellardia trixago (L.) All.	Mediterranean lineseed
Brachypodium distachyon (L.) P. Beauv.	purple false brome
Brassica nigra (L.) W.D.J. Koch	black mustard
Brassica rapa L.	field mustard
Briza maxima L.	big quakinggrass
Briza minor L.	little quakinggrass
Bromus maritimus (Piper) Hitchc.	maritime brome
Bromus carinatus Hook. & Arn. var. carinatus	California brome
Bromus diandrus Roth	ripgut brome
Bromus hordeaceus L.	soft brome
Calamagrostis nutkaensis (J. Presl) J. Presl ex Steud.	Pacific reedgrass
Calochortus albus Douglas ex Benth.	white fairy-lantern
Calochortus luteus Douglas ex Lindl.	yellow mariposa lily
Calochortus tolmiei Hook. & Arn.	Tolmie star-tulip

Calystegia purpurata (Greene) Brummitt ssp. purpurata	Pacific false bindweed
Calystegia purpurata (Greene) Brummitt ssp. saxicola (Eastw.) Brummitt	Pacific false bindweed
Camissoniopsis micrantha (Spreng.) W.L. Wagner & Hoch	miniature suncup
Cardamine oligosperma Nutt.	little western bittercress
Carex barbarae Dewey	Santa Barbara sedge
Carex brevicaulis Mack.	short stem sedge
Carex harfordii Mack.	Harford's sedge
Carex L.	sedge
Carex tumulicola Mack.	splitawn sedge
Carpobrotus chilensis (Molina) N.E. Br.	sea fig
Carpobrotus edulis (L.) L. Bolus	hottentot fig
Castilleja foliolosa Hook. & Arn.	Texas Indian paintbrush
Castilleja subinclusa Greene ssp. franciscana (Pennell) T.I. Chuang & Heckard	longleaf Indian paintbrush
Castilleja wightii Elmer	Wight's indian paintbrush
Ceanothus thyrsiflorus Eschsch.	blueblossom
Centaurea melitensis L.	Maltese star-thistle
Chenopodium californicum (S. Watson) S. Watson	California goosefoot
Chlorogalum pomeridianum (DC.) Kunth var. divaricatum (Lindl.) Hoover	wavyleaf soap plant
Chlorogalum pomeridianum (DC.) Kunth var. pomeridianum	wavyleaf soap plant
Cirsium andrewsii (A. Gray) Jeps.	Franciscan thistle
Cirsium occidentale (Nutt.) Jeps.	cobwebby thistle
Cirsium occidentale (Nutt.) Jeps. var. occidentale	cobwebby thistle
Cirsium quercetorum (A. Gray) Jeps.	Alameda County thistle
Cirsium vulgare (Savi) Ten.	bull thistle
Clarkia rubicunda (Lindl.) F.H. Lewis & M.E. Lewis	ruby chalice clarkia
Claytonia perfoliata Willd.	miner's lettuce
Clematis lasiantha Nutt.	pipestem clematis
Conium maculatum L.	poison hemlock
Cortaderia jubata (Lem.) Stapf	purple pampas grass
Cotoneaster Medik.	cotoneaster
Crassula connata (Ruiz & Pav.) A. Berger	sand pygmyweed
Cryptantha clevelandii Greene	Cleveland's cryptantha
Cryptantha muricata (Hook. & Arn.) A. Nelson & J.F. Macbr.	pointed cryptantha
Cupressus macrocarpa Hartw. ex Gord.	Monterey cypress
Cynosurus echinatus L.	bristly dogstail grass
Danthonia californica Bol.	California oatgrass
Daucus pusillus Michx.	American wild carrot
Deschampsia caespitosa (L.) P. Beauv. ssp. holciformis (J. Presl) W.E. Lawrence, orth. var.	tufted hairgrass
Deschampsia elongata (Hook.) Munro	slender hairgrass
Distichlis spicata (L.) Greene	saltgrass
Dryopteris arguta (Kaulf.) Watt	coastal woodfern
Dudleya caespitosa (Haw.) Britton & Rose	sealettuce
Dudleya farinosa (Lindl.) Britton & Rose	powdery liveforever
Elymus glaucus Buckley ssp. glaucus	blue wildrye

Epilobium ciliatum Raf.	fringed willowherb
Senecio glomeratus Desf. ex Poir.	cutleaf burnweed
Erigeron glaucus Ker Gawl.	seaside fleabane
Eriodictyon californicum (Hook. & Arn.) Torr.	California yerba santa
Eriogonum latifolium Sm.	seaside buckwheat
Eriophyllum confertiflorum (DC.) A. Gray	golden-yarrow
Eriophyllum lanatum (Pursh) Forbes var. arachnoideum (Fisch. & Avé-Lall.) Jeps.	common woolly sunflower
Eriophyllum staechadifolium Lagasca	lizard tail
Erodium botrys (Cav.) Bertol.	longbeak stork's bill
Erodium cicutarium (L.) L'Hér. ex Aiton	redstem stork's bill
Erysimum franciscanum G. Rossb.	San Francisco wallflower
Erysimum cf. menziesii (Hook.) Wettst. ssp. concinnum (Eastw.) R.A. Price	curly wallflower
Eschscholzia californica Cham.	California poppy
Eucalyptus globulus Labill.	Tasmanian bluegum
Euphorbia crenulata Engelm.	Chinese caps
Festuca californica Vasey	California fescue
Festuca idahoensis Elmer	Idaho fescue
Festuca rubra L.	red fescue
Logfia gallica (L.) Coss. & Germ	narrowleaf cottonrose
Fragaria chiloensis (L.) Mill.	beach strawberry
Fragaria vesca L.	woodland strawberry
Fritillaria affinis (Schult.) Sealy	checker lily
Galium aparine L.	stickywilly
Galium porrigens Dempster	graceful bedstraw
Gastridium phleoides (Nees & Meyen) C.E. Hubbard	nit grass
Gaultheria shallon Pursh	salal
Genista monspessulana (L.) L.A.S. Johnson	French broom
Geranium dissectum L.	cutleaf geranium
Pseudognaphalium californicum (DC.) Anderb.	California cudweed
Pseudognaphalium beneolens (Davidson) Anderb.	fragrant cudweed
Gamochaeta ustulata (Nutt.) Nesom	purple cudweed
Pseudognaphalium ramosissimum (Nutt.) Anderb.	pink cudweed
Pseudognaphalium stramineum (Kunth) Anderb.	cotton batting plant
Grindelia stricta DC. var. platyphylla (Greene) M.A. Lane	Oregon gumweed
Helenium puberulum DC.	rosilla
Heracleum maximum Bartram	cow parsnip
Heteromeles arbutifolia (Lindl.) M. Roem.	toyon
Heterotheca sessiliflora (Nutt.) Shinners ssp. bolanderi (A. Gray) Semple	sessileflower false goldenaster
Heuchera micrantha Douglas ex Lindl.	crevice alumroot
Hirschfeldia incana (L.) Lagr.-Foss.	shortpod mustard
Holcus lanatus L.	common velvetgrass
Holodiscus discolor (Pursh) Maxim.	oceanspray
Hordeum brachyantherum Nevski ssp. californicum (Covas & Stebbins) Bothmer, N. Jacobsen & Seberg	California barley
Hordeum murinum L.	mouse barley

Horkelia californica Cham. & Schltdl. ssp. californica	California horkelia
Hypochaeris glabra L.	smooth cat's ear
Hypochaeris radicata L.	hairy cat's ear
Iris douglasiana Herbert	Douglas iris
Iris macrosiphon Torr.	bowltube iris
Juncus balticus Willd. ssp. ater (Rydb.) Snogerup	Baltic rush
Juncus occidentalis Wiegand	western rush
Juncus patens E. Mey.	spreading rush
Koeleria macrantha (Ledeb.) Schult.	prairie Junegrass
Lactuca saligna L.	willowleaf lettuce
Lasthenia californica DC. ex Lindl. ssp. californica	California goldfields
Lasthenia californica DC. ex Lindl. ssp. macrantha (A. Gray) R. Chan	perennial goldfields
Lathyrus vestitus Nutt.	Pacific pea
Layia hieracioides (DC.) Hook. & Arn.	tall tidytips
Leptosiphon androsaceus Benth.	false babystars
Elymus triticoides Buckley	beardless wildrye
Ligusticum apiifolium (Nutt. ex Torr. & A. Gray) A. Gray	celeryleaf licorice-root
Ligustrum L.	privet
Linum bienne Mill.	pale flax
Lobularia maritima (L.) Desv.	sweet alyssum
Festuca perennis (L.) Columbus & J.P. Sm.	Italian ryegrass
Lonicera hispidula (Lindl.) Douglas ex Torr. & A. Gray var. vacillans A. Gray	pink honeysuckle
Lonicera involucrata (Richardson) Banks ex Spreng.	twinberry honeysuckle
Lotus corniculatus L.	bird's-foot trefoil
Acmispon glaber (Vogel) Brouillet var. glaber	deerweed
Acmispon wrangelianus (Fisch. & C.A. Mey.) D.D. Sokoloff	Chilean bird's-foot trefoil
Lupinus arboreus Sims	yellow bush lupine
Lupinus variicolor Steud.	varied lupine
Luzula comosa E. Mey.	Pacific woodrush
Madia sativa Molina	coast tarweed
Marah fabaceus (Naud.) Naud. ex Greene	California manroot
Medicago polymorpha L.	burclover
Melica torreyana Scribn.	Torrey's melicgrass
Melilotus indicus (L.) All.	annual yellow sweetclover
Microseris bigelovii (A. Gray) Sch. Bip.	coastal silverpuffs
Mimulus aurantiacus W. Curtis	sticky monkeyflower
Monardella villosa Benth. ssp. franciscana (Elmer) Jokerst	coyote mint
Monardella villosa Benth. ssp. villosa	coyote mint
Nassella lepida (Hitchc.) Barkworth	foothill needlegrass
Nassella pulchra (Hitchc.) Barkworth	purple needlegrass
Navarretia squarrosa (Eschsch.) Hook. & Arn.	skunkbush
Nemophila Nutt.	baby blue eyes
Nemophila pedunculata Douglas ex Benth.	littlefoot nemophila
Oemleria cerasiformis (Torr. & A. Gray ex Hook. & Arn.) Landon	Indian plum
Oenothera elata Kunth ssp. hookeri (Torr. & A. Gray) W. Dietr. & W.L. Wagner	Hooker's evening primrose
Oxalis albicans Kunth	white oxalis

Oxalis pes-caprae L.	Bermuda buttercup
Parapholis incurva (L.) C.E. Hubbard	curved sicklegrass
Parentucellia viscosa (L.) Caruel	yellow glandweed
Pentagramma triangularis (Kaulf.) Yatsk., Windham & E. Wollenw.	goldback fern
Perideridia kelloggii (A. Gray) Mathias	Kellogg's yampah
Phacelia californica Cham.	California phacelia
Phacelia malvifolia Cham.	stinging phacelia
Phalaris californica Hook. & Arn.	California canarygrass
Pinus radiata D. Don	Monterey pine
Piperia cf. elegans (Lindl.) Rydb. ssp. decurtata R. Morgan & Glicenstein	elegant piperia
Piperia elegans (Lindl.) Rydb. ssp. elegans	elegant piperia
Plagiobothrys chorisianus (Cham.) I.M. Johnst. var. hickmanii (Greene) I.M. Johnst.	Hickman's popcornflower
Plantago coronopus L.	buckhorn plantain
Plantago erecta Morris	dotseed plantain
Plantago lanceolata L.	narrowleaf plantain
Plantago maritima L.	goose tongue
Platystemon californicus Benth.	creamcups
Poa douglasii Nees	Douglas' bluegrass
Poa unilateralis Scribn.	San Francisco bluegrass
Polygala californica Nutt.	California milkwort
Polypodium scouleri Hook. & Grev.	leathery polypody
Polypogon interruptus Kunth	ditch rabbitsfoot grass
Polystichum munitum (Kaulf.) C. Presl	western swordfern
Prunella vulgaris L.	common selfheal
Pteridium aquilinum (L.) Kuhn var. pubescens L. Underw.	western bracken fern
Pterostegia drymarioides Fisch. & C.A. Mey.	woodland pterostegia
Quercus agrifolia Née	California live oak
Rafinesquia californica Nutt.	California plumeseed
Ranunculus californicus Benth.	California buttercup
Raphanus sativus L.	cultivated radish
Rhamnus californica Eschsch. ssp. californica	California coffeeberry
Ribes californicum Hook. & Arn. var. californicum	hillside gooseberry
Rubus spectabilis Pursh var. menziesii (Hook.) S. Watson	salmonberry
Rubus ursinus Cham. & Schltdl.	California blackberry
Rumex acetosella L.	common sheep sorrel
Rumex crispus L.	curly dock
Rumex crassus Rech. f.	maritime willow dock
Rumex salicifolius J.A. Weinm.	willow dock
Sagina maxima A. Gray ssp. crassicaulis (S. Watson) Crow	stickystem pearlwort
Sambucus nigra L. ssp. caerulea (Raf.) R. Bolli	blue elderberry
Sambucus racemosa L.	red elderberry
Sanicula arctopoides Hook. & Arn.	footsteps of spring
Sanicula bipinnatifida Douglas ex Hook.	purple sanicle
Sanicula crassicaulis Poepp. ex DC.	Pacific blacksnakeroot
Clinopodium douglasii (Benth.) Kuntze	yerba buena
Isolepis carinata Hook. & Arn. ex Torr.	keeled bullrush

Scrophularia californica Cham. & Schltdl.	California figwort
Sidalcea malviflora (DC.) A. Gray ex Benth. ssp. malviflora	dwarf checkerbloom
Silene gallica L.	common catchfly
Silybum marianum (L.) Gaertn.	blessed milkthistle
Sisyrinchium bellum S. Watson	western blue-eyed grass
Maianthemum stellatum (L.) Link	starry false lily of the valley
Solanum douglasii Dunal	greenspot nightshade
Solidago velutina DC. ssp. californica (Nutt.) Semple	California goldenrod
Solidago spathulata DC.	dune goldenrod
Sonchus asper (L.) Hill	spiny sowthistle
Sonchus oleraceus L.	common sowthistle
Spergularia macrotheca (Hornem.) Heynh. var. macrotheca	sticky sandspurry
Stachys rigida Benth. var. quercetorum (A. Heller) G.A. Mulligan & D.B. Munro	rough hedgenettle
Stachys bullata Benth.	California hedgenettle
Stellaria media (L.) Vill.	common chickweed
Symphoricarpos albus (L.) S.F. Blake	common snowberry
Toxicodendron diversilobum (Torr. & A. Gray) Greene	Pacific poison oak
Trifolium albopurpureum Torr. & A. Gray	rancheria clover
Trifolium dubium Sibth.	suckling clover
Trifolium hirtum All.	rose clover
Trifolium willdenovii Spreng.	tomcat clover
Trisetum canescens Buckley	tall trisetum
Triteleia laxa Benth.	Ithuriel's spear
Umbellularia californica (Hook. & Arn.) Nutt.	California laurel
Vaccinium ovatum Pursh	California huckleberry
Verbena lasiostachys Link	western vervain
Vicia americana Muhl. ex Willd. ssp. americana	American vetch
Vicia sativa L.	garden vetch
Vicia tetrasperma (L.) Schreb.	lentil vetch
Viola pedunculata Torr. & A. Gray	Johnny-jump-up
Festuca bromoides L.	brome fescue
Festuca myuros L.	rattail fescue
Wyethia angustifolia (DC.) Nutt.	California compassplant
Zeltnera muehlenbergii (Griseb.) G. Mans.	Muhlenberg's centaury
