# Supplementary material for: Local patterns of diversity in California northern coastal scrub
Source: Ecol Evol. 2018 Jun 27;8(15):7250–60. doi: 10.1002/ece3.4104 (PMC6106371; doi:10.1002/ece3.4104)
Supplement: Supplementary file 7 [file ECE3-8-7250-s007.pdf]

Soils analysis. Western Agricultural Laboratories, Modesto, California.

Wrubel, E. Parker, V.T. (2018). Local patterns of diversity in northern coastal scrub of California. *Ecology and Evolution*.

			Oranic Matter		Phosphorus		Potassium	Magnesium	Calcium	Sodium	pH			Cation Exchange Capacity			Percent Cation Saturation (Computed)			
**Site**	**Continentality**	**Species richness**	**% Rating**	**ENR lbs/Acre**	**P1 (Weak Bray) ppm**	**$NaHCO_3P$ (Olsen) ppm**	**K ppm**	**Mg ppm**	**Ca ppm**	**Na ppm**	**Soil pH**	**Buffer Index**	**H meq/100g**	**CEC meq/100g**	**K %**	**Mg**	**Ca**	**H**	**Na**	**Sulfur SO_4-S ppm**
1	coast	31	2.5	80	15	19	146	357	1340	83	6.4	6.9	1	11.4	3.3	25.8	58.7	9	3.2	11
2	coast	32	5.2	133	8	15	396	777	1682	156	6.6	1.1	17.5	5.8	36.5	47.9	6	3.9		13
3	coast	19	5.1	132	29	29	185	612	1634	327	6.2	6.7	2.1	17.1	2.8	29.4	47.6	12	8.3	17
4	coast	22	4	109	6	12**	271	614	1051	249	5.6	6.6	3.7	15.8	4.4	32	33.3	23.5	6.9	25
5	inland	11	4	110	11	17**	332	887	1943	42	6	6.6	3.2	21.2	4	34.4	45.7	15	0.9	10
6	inland	15	*	*	*	*	*	*	*	*	*	*	*	*	*	*	*	*	*	*
7	inland	10	*	*	*	*	*	*	*	*	*	*	*	*	*	*	*	*	*	*
8	coast	21	5.9	147	8	19**	431	825	1784	218	5.9	6.6	3.6	21.4	5.2	31.7	41.7	17	4.4	15
9	coast	23	6.8	166	6	15**	435	697	1625	136	5.8	6.6	3.6	19.2	5.8					12
10	inland	20	8.5	199	19	25**	167	819	1632	103	5.8	6.6	3.7	19.4	2.2	34.6	41.9	19	2.3	9
11	coast	24	*	*	*	*	*	*	*	*	*	*	*	*	*	*	*	*	*	*
12	coast	23	6.3	156	8	20	474	806	1621	456	6.4	6.8	1.8	19.7	6.2	33.7	41.1	9	10.1	35
13	coast	28	7.5	181	6	14	319	1845	3684	632	6.7	1.7	38.9	2.1	39	47.3	4.5	7.1		17
14	coast	34	5.9	148	13	18**	212	426	946	89	5.7	6.7	2.4	11.6	4.7	30.2	40.8	21	3.4	17
15	coast	16	4.9	127	12	16	355	786	1714	113	6.3	6.7	1.9	18.3	5	35.2	46.6	10.5	2.7	11
16	coast	22	4.1	112	15	9	294	632	1320	279	6.4	6.8	1.4	15.1	5	34.4	43.6	9	8	24
17	coast	25	3.1	92	17	17**	212	450	639	169	5.4	6.6	3.3	11.4	4.7	32.4	27.9	28.5	6.4	19
18	inland	12	5.1	132	8	16	458	973	2766	35	6.6	1.5	24.6	4.8	32.5	56.1	6	0.6		15
19	coast	22	6.3	157	4	15**	433	1055	1564	424	5.7	6.5	5.2	24.6	4.5	35.3	31.7	21	7.5	19
20	coast	19	5.9	148	4	13**	385	1064	1901	222	5.6	6.4	6.2	26.4	3.7	33.2	36	23.5	3.7	10
21	inland	22	6.2	154	9	16**	646	427	1775	41	5.6	6.5	4.4	18.6	8.9	18.9	47.7	23.5	1	20
22	coast	25	*	*	*	*	*	*	*	*	*	*	*	*	*	*	*	*	*	*
23	inland	13	5.3	135	4	14**	310	613	2643	57	5.8	6.5	4.5	23.8	3.3	21.2	55.4	19	1	9
24	coast	32	*	*	*	*	*	*	*	*	*	*	*	*	*	*	*	*	*	*
25	inland	17	6.3	155	11	23	494	1225	3888	25	6.2	6.6	4.2	35.1	3.6	28.7	55.3	12	0.3	10
26	coast	22	5	131	9	24	137	450	2272	110	6.4	6.8	1.6	17.4	2	21.2	65	9	2.7	15
27	inland	20	12.8	286	21	31	558	448	3967	66	6.8	0.8	26	5.5	14.2	76.2	3	1.1		8

* No data
** NaHCO3-P unreliable at this soil pH
